# Elevated lactate levels and impaired lactate clearance during extracorporeal life support (ECLS) are associated with poor outcome in cardiac surgery patients

**DOI:** 10.1371/journal.pone.0278139

**Published:** 2022-11-28

**Authors:** René Rissel, Sascha Koelm, Markus Schepers, Daniel-Sebastian Dohle, Joerg Albers, Mehmet Oezkur, Marc Kriege, Marc Bodenstein

**Affiliations:** 1 Department of Anaesthesiology, Medical Centre of the Johannes Gutenberg-University, Mainz, Germany; 2 Institute of Medical Biostatistics, Epidemiology and Informatics (IMBEI), University Medical Center, Johannes Gutenberg University, Mainz, Germany; 3 Department of Cardiothoracic and Vascular Surgery, University Hospital of Johannes Gutenberg University Mainz, Mainz, Germany; Albert Einstein College of Medicine, UNITED STATES

## Abstract

The use of extracorporeal life support (ECLS) as part of cardio-circulatory support has increased rapidly in recent years. Severe hyperlactatemia is not uncommon in this group of patients. Lactate peak concentrations and lactate clearance have already been identified as independent marker for mortality in critical ill patients without mechanical device support. The aim of this study was to determine a supposed correlation between the variables lactate peak concentration and clearance in the blood and mortality in the ECLS context. Therefore, a total of 51 cardiac surgery ICU patients with ECLS therapy were included in this retrospective, clinical observational study (survivors n = 23; non-survivors n = 28). Lactate measurement was performed before, during and after ECLS therapy. Further, common ICU scores (SAPSII, SOFA, TISS28), the rates of transfusion and the different vasopressor therapies will be compared. Significant elevated peak lactate levels and poor lactate clearance were associated with higher mortality during ECLS therapy (p < 0.001). Deceased patients had higher SAPSII scores (p < 0.001), received more transfusions (p < 0.001) and presented with higher rates of epinephrine (p < 0.001). In conclusion, hyperlactatemia during ECLS therapy is a time sensitive emergency. Lactate cannot be cleared in all patients. Reversible causes should be explored and treated. In cases where the cause is irreversible, the prognosis of elevated lactate concentrations and reduced clearance is very poor.

## Introduction

Severe hyperlactatemia (> 10.0 mmol/L) is a predictor of poor clinical outcome for unselected critical ill patients (a.e. sepsis or trauma patients). Data in patients with acute cardiac conditions are controversial due to the fact that the role of lactate clearance in acute cardiac settings is so far not assessed, basically for lack of data in this scenario [1]. Peak lactate levels and no marked lactate clearance within 12 hours predict a high ICU mortality [2, 3]. The causes of perioperative hyperlactatemia are varied and include hypoxic and non-hypoxic causes [4]. Refractory cardiogenic shock (RCS), defined as cardiac and circulatory failure resulting in organ hypoperfusion unresponsive to conventional medical therapies, may be one cause of severe hyperlactatemia. Thereby, RCS can occur after cardiac operations as postcardiotomy cardiogenic shock, extracorporeal cardiopulmonary resuscitation (eCPR) or complicated transcatheter valve implantation [5]. Early aggressive management with initiation of veno-arterial extracorporeal life support (VA-ECLS) represents a valuable therapeutic option to stabilize patients’ condition in medically RCS [6, 7]. The incidence of this mechanical circulatory support evolved in the last years [8]. Today’s technique of VA-ECLS is safer, miniaturized available and more durable with fewer complications [9]. Limited data are available on the clinical significance of lactate concentration and lactate clearance as a short-term prognostic tool for patients in RCS with VA-ECLS therapy. We therefore aimed to evaluate the association between peak lactate concentration and lactate clearance with ICU mortality in cardiac surgery patients with VA-ECLS therapy in RCS in a first unspecific statistical analysis.

## Methods

The study was approved by the appropriate Ethics authority (Ethikkommission der Landesärztekammer Rheinland-Pfalz; 2022–16288; 10/01/2022) and adheres to the applicable CONSORT guidelines. The need for written/verbal consent was waived by the ethics committee.

In this retrospective, observational, single center study from a university hospital department with one cardiac surgery ICU during the study period between 01. January 2020 and 28. February 2021, we screened for patients with unselected causes of RCS and VA-ECLS. Clinical details were retrospectively extracted from a prospective record of our patient data system (COPRA Live, version 6.1, Germany).

### Patient’s characteristics

Demographic characteristics (a.e. age, sex, body mass index), medical/surgical history and routine laboratory findings were collected. Further, common ICU scores (a.e. SAPSII, SOFA and TISS-28), necessary organ replacement and support (a.e. intraaortic ballon pump or continuous renal replacement), transfusion frequency and the length of ICU stay were extracted from our patient data system. To minimize bias, we exclude patients with respiratory failure and undergoing veno-venous extracorporeal membrane oxygenation (ECMO). Also, we excluded patients who had to be transferred to a specialized center for heart transplantation or long-term mechanical cardiac support implantation. Further, only patients from the cardiac surgery ICU with a complete set of data were included.

### Parameter setting for lactate as an outcome parameter

Lactate concentrations were measured routinely every 4 hours from arterial blood gas samples. For this protocol, we focused on the lactate concentration before the ECLS had commenced the peak and minimum lactate concentrations during ECLS therapy and the time spent in defined lactate levels in hours (a.e. hours within lactate levels ≤ 5.0 mmol/L, 5.1–10.0 mmol/L, 10.1–15.0 mmol/L, 15.1–20.0 mmol/L and ≥ 20.1 mmol/L). Further, we measured the lactate level after weaning of VA-ECLS.

### Type of VA-ECLS

In this study, we enrolled patients with central (a.e. aortal or subclavian artery) or peripheral (femoral artery) VA-cannulation. The cannulation was performed either in the operating room (arterial cannula at ventral position) or at bedside on the ICU using the Seldinger’s technique (arterial cannula at peripheral position). Cannula sizes were selected according to the patient’s body weight, with arterial cannula 15–20 F and venous cannula 20–26 F. In the case of extremity ischemia, detected by eight hourly Doppler examinations, an additional reperfusion cannula was implanted for the affected extremity. Therefore, Color Doppler is a valuable bed-side, fast and accepted diagnostic method in detecting pathology of the lower limb arteries. Changes in hemodynamic indices as the pulse wave form are of help for identifying disorders that affect tissue perfusion (a.e. stenosis, arterial/venous thrombus) [10, 11]. Further, all patients received an activated clotting time (ACT) guided (120 to 150 seconds) anticoagulation intravenously with heparin. The oxygenator was set with oxygen flow and sweep air gas according to arterial blood gas analysis results. VA-ECLS flow was set to maintain proper organ oxygenation and perfusion. This was controlled with repetitive laboratory tests and daily redefined hemodynamic and respiratory targets (a.e. cardiac index ≥ 2.2 L/min/m^2^, mean arterial blood pressure between 60–75 mmHg, hemoglobin levels ≥ 8 g/dl, a mixed venous saturation level around 70%, body temperature between 36 to 37.5°C). During VA-ECLS, all patients were sedated with one or more anesthetics (a.e. sufentanil, propofol, ketamine, midazolam or dexmedetomidine) to reach a sedation scale from RASS 0 to RASS -2 (RASS = Richmond Agitation and Sedation Scale). Awake VA-ECLS was possible with peripheral cannulation. VA-ECLS weaning started in absence of any hemodynamic abnormalities and no foci of bleeding. VA-ECLS flow was reduced stepwise in accordance to an individual created weaning plan under transthoracic/transesophageal echocardiography and invasive hemodynamic monitoring, including pulmonary artery catheter (PAC) (a.e. preserved left ventricular ejection fraction, no left ventricular distension, no right ventricular failure) when lactate levels decreased and remained stable. A renewed increase in lactate levels was first counteracted with a VA-ECLS pump flow increase. Secondly, the weaning plan and the predefined target values ​​(a.e. cardiac index ≥ 2.2 L/min/m^2^, mean arterial blood pressure between 60–75 mmHg, hemoglobin levels ≥ 8 g/dl, a mixed venous saturation level around 70%, body temperature between 36 to 37.5°C) were then checked and adjusted. The transthoracic/transesophageal echocardiography examination of the heart was carried out to support this (a.e. to rule out valve pathologies, regional wall movement abnormalities or cardiac tamponade). To ensure left ventricular (LV) unloading an Impella implantation was performed. The indications to ensure LV-unloading were a distended and hypocontractile LV, a stagnation of blood in the LV and/or above the aortic valve when seen on echo or an elevated pulmonary capillary wedge pressure greater than 25 mmHg measured with a pulmonary artery catheter. Further, VA-ECLS related complications (a.e. hemolysis, limb amputation due to ischemia, stroke/intracerebral hemorrhage (ICH)), rethoracotomy or additional manual CPR were compared.

### Statistical analysis

Statistical analyses were performed from the Institute of Medical Biostatistics, Epidemiology and Informatics (IMBEI), University Medical Center of the Johannes Gutenberg University Mainz using R (Version 4.1.2; R core team, Austria). All datasets were collected and analyzed using Microsoft Excel (Version 14.0; Microsoft, USA). Categorical variables were expressed as frequency distributions and were tested with Fisher’s exact test or Pearson’s Chi-square test. Univariate comparisons between the groups on categorical variables were performed using the Fisher’s exact test or Pearson’s Chi-square test as appropriate. Continuous variables were evaluated for normality using the Shapiro-Wilk test. As data was non-normally distributed, Wilcoxon rank sum test was used and expressed as the arithmetic mean ± standard deviation. The predictive accuracy of lactate levels with respect to the maximum lactate concentration was assessed using the area under the receiver operating characteristic (ROC) curve with a maximized Youden Index. A statistical significance was set at a value of 5% (p < 0.05).

## Results

**Fig 1:**
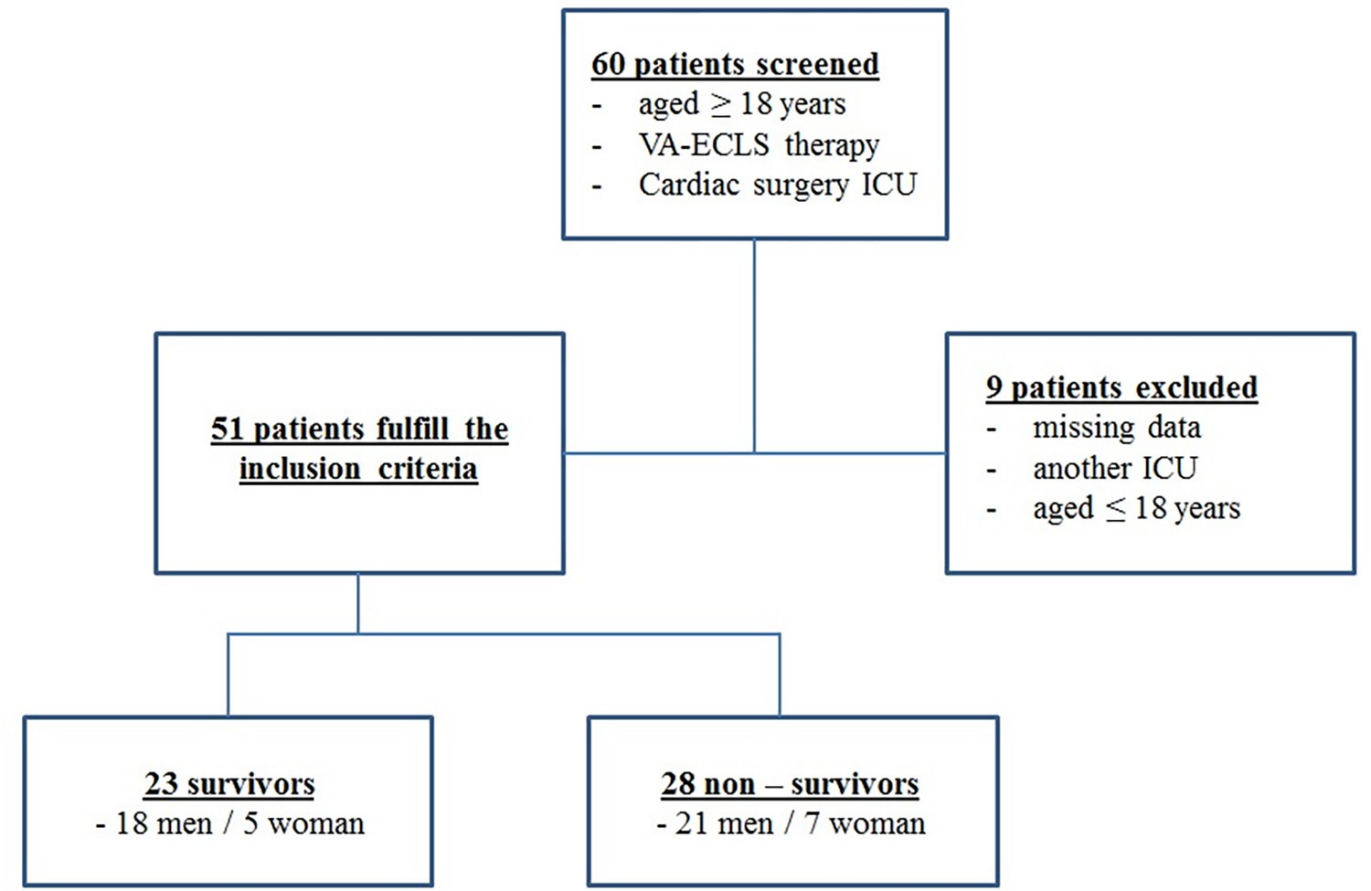
Study design.

60 patients were screened for this study (Fig 1). A total of 51 adult patients who underwent VA-ECLS therapy were enrolled in this study. Nine patients were excluded due to missing data or discharging to another ICU. The cohort was divided into survivors (n = 23, mean age 66.3 ± 9.5) and non-survivors (n = 28, mean age 61.0 ± 13.7). The survivors consisted of 18 men and 5 women and the non-survivors consisted of 21 men and 7 women, respectively.

In both groups, nine patients did not receive a cardiac surgery procedure (Table 1). In this case, VA-ECLS implantation was performed as eCPR due to complicated transcatheter valve repair (one patient in each group; Table 1), frustrated percutaneous coronary intervention (PCI; 13% survivors vs. 17.8% non-survivors; Table 1), massive pulmonary embolism (13% survivors vs. 7% non-survivors; Table 1) or other life-threatening circumstances (one patient in each group; Table 1).

**Table 1:** Patient’s characteristics.

Characteristics	Survivors	Non-survivors	P value
	(N = 23)	(N = 28)	
**Age (years)**	66.3±9.5	61.0±13.7	0.125
**Sex (male/female)**	18/5	21/7	0.686
**Body mass index (kg/m^2^)**			
<18.5	0	0	/
18.5–24.9	6 (26%)	14 (50%)	0.087
25–29.9	11 (48%)	7 (25%)	0.095
30–34.9	4 (17%)	3 (11%)	0.505
35–39.9	1 (4.5%)	2 (7%)	0.693
≥40	1 (4.5%)	2 (7%)	0.693
**Pre-existing illness**			
Coronary Heart Disease	14 (60.9%)	18 (64.3%)	0.812
Myocardial infarction	6 (26.1%)	7 (25%)	0.940
Cardiac valvulopathy	11 (47.8%)	10 (35.7%)	0.393
Heart failure	8 (34.8%)	7 (25%)	0.457
Diabetes mellitus	11 (47.8%)	15 (53.6%)	0.694
Smoking	6 (26.1%)	10 (35.7%)	0.473
COPD	1 (4.4%)	5 (17.9%)	0.145
Pulmonary emphysema	1 (4.4%)	2 (7.1%)	0.693
Chronic kidney disease	2 (8.7%)	5 (17.9%)	0.357
Liver disease	0 (0%)	1 (3.6%)	0.386
**Major preoperative diagnosis**			
Pulmonary embolism + eCPR	3 (13%)	2 (7%)	0.497
TAVR + eCPR	1 (4.4%)	1 (3.6%)	0.910
PCI + eCPR	3 (13%)	5 (17.8%)	0.265
Cardiac decompensation due			
to aortic valve stenosis III°	4 (17%)	2 (7%)	0.270
to aortic valve insufficiency III°	2 (8.7%)	1 (3.6%)	0.457
to tricuspid valve insufficiency III°	1 (4.4%)	0	0.287
to mitral valve stenosis III°	1 (4.4%)	0	0.287
to mitral valve insufficiency III°	1 (4.4%)	1 (3.6%)	0.910
to STEMI	3 (13%)	9 (32%)	0.116
to STEMI + ventricle rupture	1 (4.4%)	2 (7%)	0.693
Aortic root abscess	1 (4.4%)	0	0.287
Type A aortic dissection	0	4 (14%)	0.064
Endocarditis	1 (4.4%)	5 (17.9%)	0.145
Others	1 (4.4%)	1 (3.6%)	0.910
**Operation**			
None	9 (39%)	9 (32%)	0.797
CABG	5 (21.7%)	2 (7.1%)	0.140
Valve surgical procedures	2 (8.7%)	5 (17.9%)	0.357
Combination (CABG + valve)	7 (30.4%)	12 (42.9%)	0.372
**Length of ICU stay (days)**	23.0±35.5	13.5±14.8	0.670
**VA-ECLS therapy (hours)**	103.2±94.1	187.3±165.5	0.094
**VA-ECLS implantation**			
CPB weaning failure after surgery	3 (13.0%)	10 (35.7%)	0.069
postoperative	13 (56.5%)	10 (35.7%)	0.144
eCPR	7 (30.4%)	8 (28.5%)	0.895
**VA-ECLS cannulation**			
central	11 (47.8%)	17 (60.7%)	0.368
peripheral	12 (52.2%)	11 (39.3%)	0.368
**Vasopressors**			
Norepinephrine	23 (100%)	28 (100%)	1.000
Epinephrine	13 (56.5%)	27 (96.4%)	< 0.001
Dobutamine	16 (69.5%)	22 (78.5%)	0.475
Milrinone	12 (52.2%)	12 (42.8%)	0.518
Vasopressin	8 (34.7%)	5 (17.8%)	0.176
Levosimendan	11 (47.8%)	16 (57.1%)	0.518
**Organ replacement/support**			
Impella	0 (0%)	3 (10.7%)	0.114
IABP	4 (17.3%)	7 (25.0%)	0.524
CRRT	15 (65.2%)	24 (85.7%)	0.092
**Transfusion**			
Red blood cell concentrates	26.6±29.6	29.4±36.6	0.007
Fresh frozen plasma	22.5±27.5	27.3±31.8	0.005
Thrombocytes concentrates	7.1±12.3	12.2±12.4	0.009
**Complications**			
Rethoracotomy	7 (30.4%)	11 (39.3%)	0.522
Manual CPR	4 (17.4%)	6 (21.4%)	0.731
Intracerebral hemorrhage	1 (4.4%)	4 (14%)	0.247
Stroke	1 (4.4%)	4 (14%)	0.247
Limb amputation	2 (8.8%)	1 (3.6%)	0.457
Hemolysis	5 (21.7%)	10 (35.7%)	0.286

Coronary artery bypass graft (CABG) was performed at five survivors and two non-survivors (21.7% vs. 7.1%; Table 1), whereas valve surgical procedures were more performed in the non-survivor group compared to survivors (17.9% vs. 8.7%; Table 1). Combination procedures (CABG + valve) received seven survivor and 12 non-survivors (Table 1). The detailed major preoperative diagnosis and further information about further demographic characteristics are clearly summarized in Table 1. The average duration of VA-ECLS therapy in hours did not significantly differs between survivors and non-survivors (103.2 ± 94.1 vs. 187.3 ± 165.5). The VA-ECLS implantation site and type of cannulation showed no statistical differences. In both groups, CRRT was the most used organ replacement therapy (65.2% survivors and 85.7% for non-survivors; Table 1). The IABP was used more frequently in the non-survivor group without being significant (25.0% vs. 17.3%; Table 1). Three non-survivors were supported by an Impella (10.7%). Epinephrine was used more frequently in the non-survivor group (96.4% vs. 56.5%; p < 0.001; Table 1). Red blood cell concentrates, fresh frozen plasma and thrombocytes concentrates were more frequently transfused in the non-survivor group (p < 0.05; Table 1). The complications in both groups are further summarized in Table 1. Compared to the non-survivors, survivors had a lower last SOFA score (7.5 ± 2.9 vs 18.1 ± 4.5; p < 0.001; Table 2) and a lower overall minimum SOFA score (5.2 ± 2.1 vs. 11.8 ± 5.4; p < 0.05; Table 2).

**Table 2:** ICU scores.

Score	Survivors	Non-survivors
	(N = 23)	(N = 28)
SOFA-First	11.6±4.8	12.5±5.7
SOFA-Last	7.5±2.9	18.1±4.5*
SOFA-Minimum	5.2±2.1	11.8±5.4^#^
SOFA-Maximum	13.8±4.6	18.3±4.6
SAPS II-First	69.2±17.3	65.4±17.7
SAPS II-Last	53.0±23.0	86.2±12.4*
SAPS II-Minimum	40.3±18.5	52.2±19.0^#^
SAPS II-Maximum	84.5±10.4	94.3±8.6*
TISS 28-First	44.1±10.7	39.6±12.2
TISS 28-Last	32.8±9.0	48.8±8.4*
TISS 28-Minimum	27.3±6.6	32.0±10.5
TISS 28-Maximum	53.6±6.9	55.4±6.0

* p < 0.001

# p < 0.05.

Additionally, survivors showed a lower last, overall minimum and maximum SAPSII score (SAPSII last: 53.0 ± 23.0 vs. 86.2 ± 12.4; p < 0.001; SAPSII minimum: 40.3 ± 18.5 vs. 52.2 ± 19.0; p < 0.05; SAPSII maximum: 84.5 ± 10.4 vs. 94.3 ± 8.6; p < 0.01; Table 2). Further, non survivors presented with a higher last TISS28- score compared to survivors (48.8 ± 8.4 vs. 32.8 ± 9.0; p < 0.001; Table 2). All the ICU scores are summarized in detail in Table 2. The overall minimum and last thrombocytes count remained lower in the non-survivor’s group (Thrombocytes last: 84 ± 47 vs. 176 ± 80; p < 0.001; Thrombocytes minimum: 47 ± 29 vs. 67 ± 42; p < 0.05; Table 3).

**Table 3:** Laboratory parameters.

Laboratory	Survivors	Non-survivors
	(N = 23)	(N = 28)
Bilirubin-First [mg/dl]	1.3±1.1	2.0±1.6
Bilirubin-Last [mg/dl]	3.4±6.8	21.1±19.9*
Bilirubin-Minimum [mg/dl]	0.8±1.0	1.6±1.2*
Bilirubin-Maximum [mg/dl]	6.5±7.6	27.2±20.6*
Creatinine-First [mg/dl]	1.6±1.2	1.7±1.0
Creatinine-Last [mg/dl]	1.5±0.6	3.1±8.2
Creatinine-Minimum [mg/dl]	1.0±0.3	1.0±0.5
Creatinine-Maximum [mg/dl]	2.5±1.3	4.3±8.0
CRP-First [mg/L]	53.2±62.3	82.5±95.3
CRP-Last [mg/L]	111.1±76.0	172.4±117.3
CRP-Minimum [mg/L]	32.3±35.0	37.4±42.6
CRP-Maximum [mg/L]	255.1±107.7	263.4±129.7
PCT-First [ng/ml]	23.6±26.8	15.8±17.2
PCT-Last [ng/ml]	6.4±13.2	13.2±10.5*
PCT-Minimum [ng/ml]	5.9±3.4	5.1±5.3^#^
PCT-Maximum [ng/ml]	35.4±32.5	35.2±29.4
Thrombocytes-First [mcL]	217±106	173±87
Thrombocytes-Last [mcL]	176±80	84±47*
Thrombocytes-Minimum [mcL]	67±42	47±29^#^
Thrombocytes-Maximum [mcL]	245±104	216±85
pH-Minimum	7.19±0.09	7.08±0.15^#^
Lactate pre VA-ECLS [mmol/L]	8.63±4.72	10.34±6.13
First lactate at VA-ECLS [mmol/L]	8.59±4.71	11.28±6.11
Last lactate at VA-ECLS [mmol/L]	1.56±0.66	13.71±8.96*
Lactate post VA-ECLS [mmol/L]	1.49±0.71	11.4±8.03*
Lactate maximum [mmol/L]	12.41±12.71	19.70±6.64*
Lactate minimum [mmol/L]	1.32±1.31	2.61±2.61

* p < 0.001

# p < 0.05.

**Fig 2:**
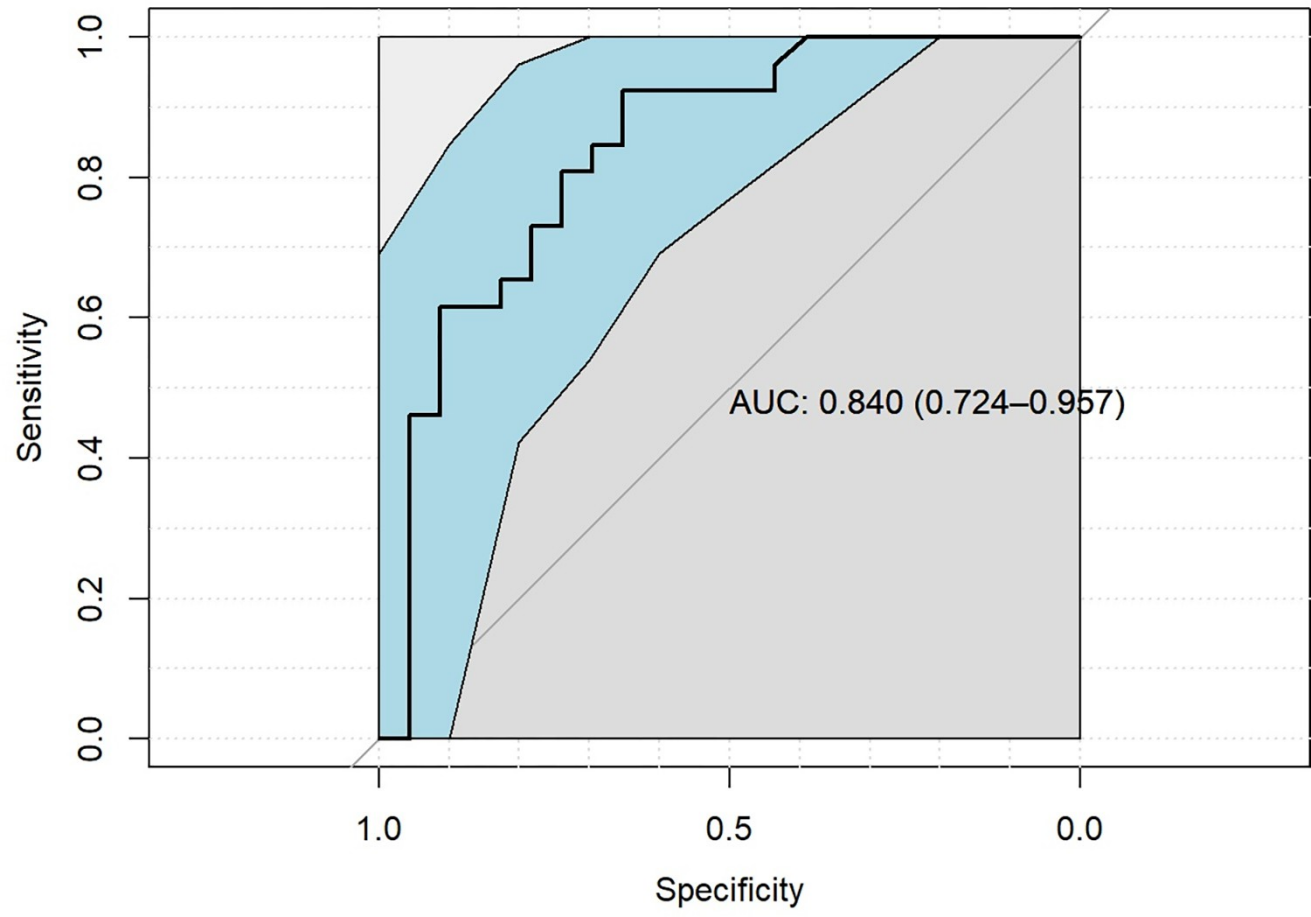
ROC curve peak lactate level.

Likewise, the non-survivors had a higher level of the last bilirubin (21.1 ± 19.9 vs. 3.4 ± 6.8; p < 0.001; Table 3), an elevated overall minimum and maximum bilirubin level (1.6 ± 1.2 vs. 0.8 ± 1.0 and 27.2 ± 20.6 vs. 6.5 ± 7.6; p < 0.001; Table 3). Compared to the survivors, the non-survivors had a lower overall pH minimum (7.08 ± 0.15 vs. 7.19 ± 0.09; p < 0.05; Table 3). Lactate levels pre VA-ECLS initiation were elevated but showed no significant intergroup differences as well as the first lactate levels measured after beginning on VA-ECLS (Table 3). Compared to the non-survivors, survivors had a lower last lactate level at VA-ECLS (1.56 ± 0.66 vs. 13.71 ± 8.96; p < 0.001; Table 3). Additionally, survivors had a significant lower post VA-ECLS weaning lactate level (1.49 ± 0.71 vs. 11.4 ± 8.03; p < 0.001; Table 3). The overall peak lactate level was elevated in the non-survivor group (19.70 ± 6.64 vs. 12.41 ± 12.71; p < 0.001; Table 3). Table 3 summarized in detail all the measured lactate values. The maximum lactate level in patients with a critical lower limb ischemia and necessary amputation was elevated in the non-survivor group compared to the survivors without being significant (15.3 ± 0.0 vs. 7.8 ± 0.41; p > 0.368). In the receiver operating characteristic (ROC) curve the peak lactate level was a good choice to distinguish between survivors and non-survivors (area under the curve (AUC): 0.840); Fig 2). An optimal cut-off point to discriminate between survivors and non-survivors could be a lactate level of 11.5 mmol/L (accuracy: 0.7959; sensitivity: 0.9231; specificity: 0.6522).

**Fig 3:**
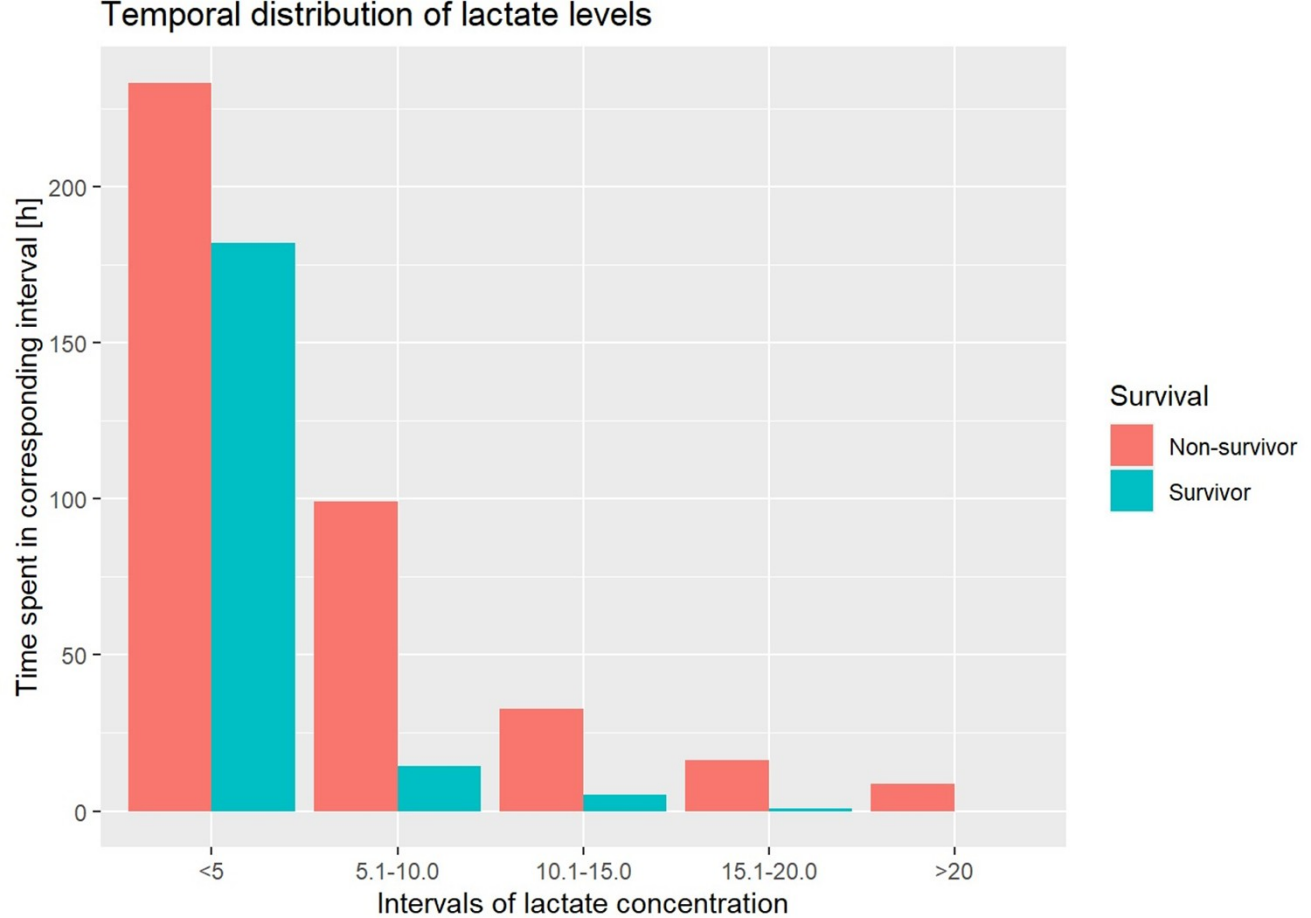
Temporal distribution of lactate levels.

Likewise, survivors presented with significant lower hours compared to non-survivors in all the prespecified lactate intervals (<5.0: 90.89 ± 97.79 vs. 116.61 ± 160.17; 5.1–10.0: 7.15 ± 7.63 vs. 51.38 ± 70.32; 10.1–15.0: 2.67 ± 6.57 vs. 16.38 ± 18.79; 15.1–20.0: 0.41 ± 1.07 vs. 8.07 ± 10.69; >20.1: 0.0 ± 0.0 vs. 4.42 ± 7.19; p < 0.001 for all the intervals; Fig 3).

## Discussion

The present retrospective study investigated the role of lactate clearance and peak lactate levels on VA-ECLS therapy on survival due to unselected RCS. Main findings of this study are:

1. The temporal distribution to elevated serum lactate levels (> 5.0 mmol/L) is associated with higher mortality on VA-ECLS therapy.
2. The lactate peak concentration was increased in the non-survivor group and could be a factor to distinguish between survival and non-survival.
3. Pre VA-ECLS therapy lactate levels were elevated but showed no intergroup differences.

The use of ECLS has been improved and generalized in clinical practice over the last years [8]. ECLS support is a final treatment modality for patients in the RCS. New developed devices that are smaller and simpler in handling offer a prolonged ECLS support. Nevertheless, patients with ECLS therapy show mortality rates between 40 to 60% [9]. As already known from septic patients with similar high mortality rates, deceased patients in our study presented with higher ICU scores [12, 13]. Due to this fact, further independent risk factors and measurable values that might predict survival and identify the patients who could most benefit from ECLS needed to be investigated. SAPSII score has already been investigated with valuable information about prediction of survival and weaning from VA-ECLS in the past [14].

In our study, the demographic basics and pre-clinical history did not differ significantly. However, attention should be paid to that acute myocardial infarction (AMI) with complications (a.e. STEMI with/without rupture of the ventricular septum), typ A aortic dissection and patients with endocarditis were more frequently in the non-survivor group. Under these critical circumstances, VA-ECLS use is known to be associated with higher mortality and well described for AMI with mechanical complications [15].

Nevertheless, ECLS offers a total hemodynamic support with fully oxygenated blood for those patients with RCS. Blood lactate is a well know diagnosis and prognosis biomarker in critical ill patients [2]. Especially in context with a presence tissue hypoxia and hypoperfusion [2, 16]. Lactate levels are placed in the center of different resuscitation evidence-based therapy protocols [17, 18]. However, the complex metabolism and clearance of lactate and its confounders, especially under ECLS remain unclear and lack of evidence. Regarding the third point of our results, previous studies confirmed elevated lactate levels in survivors and deceased patients before ECLS therapy [19, 20]. A critical cut-off lactate level above 12.6 mmol/L was associated with higher mortality [19]. Same results are known from septic patients. Initially increased serum lactate levels are able to risk-stratify patients with severe sepsis [21]. In both, elevated lactate levels reflect the reduced tissue perfusion and organ supply with oxygen due to cardiac failure. Anaerobic metabolism occurs and is responsible for the hypoxic hyperlactatemia [22]. In conclusion, patients in acute medicine benefit from serial blood lactate measurements [23]. An early initiation of ECLS therapy at lower serum lactate levels for patients with RCS could improve the outcome [6, 24–26]. Further and larger study cohorts are necessary to understand and investigate the role of elevated lactate levels on mortality for patients with RCS before initiating an ECLS therapy. Especially the contribution of non-hypoxic causes of hyperlactatemia needs to be addressed [27].

Peak serum lactate levels were analyzed to predict survival of critical ill patients in the past. For septic patients, higher lactate levels were the best threshold to predict 28-day mortality [27, 28]. After starting with the sepsis bundles, the six-hour lactate level above 2 mmol/L had the highest sensitivity for predicting mortality [27]. Another study claimed a lactate level of 4 mmol/L as a strong predictor of survival for septic patients [29]. Similar results were seen in patients with acute myocardial infarction and the need of ECLS. Lactate-increase greater than 4 mmol/L seems to be a helpful threshold to trigger the timely onset of ECLS-therapy for these patients [30]. In our study, deceased patients had significant elevated peak serum lactate level. Here, a suggested cut-off point to discriminate between survivors and non-survivors could be a peak lactate level of 11.5 mmol/L. No studies investigated the role of the peak serum lactate level on mortality for patients in RCS with ECLS therapy. Septic patients and patients suffering from RCS underlie different pathologies. But based on the findings from septic research and due to the fact that the ECLS support could not achieve a decrease of lactate in the non-survivors in our study, the peak lactate level could be a predictor of mortality for this cohort. Further investigations must address this topic.

Among all the previous lactate metrics, lactate clearance as a “dynamic” parameter in critical ill patients seems to be the most investigated in literature. Especially patients with septic shock were in the focus of these investigations [23, 27–29]. Thereby, clearance is defined as the removal of a substance from blood [31]. Changes in the serum lactate level can be associated with ongoing alterations in production, excretion, metabolism of lactate or drug interactions [31]. Due to these complex lactate interactions several lactate clearance “indices” has been described: “lactate delayed”, “lactate clearance”, “LacScale”, “normalized lactate load” and “lactate load” [26, 32, 33]. In our study, we compared the time spent in prespecified lactate intervals between survivors and non-survivors. Spending more hours in a higher lactate interval reflects an impaired lactate clearance. As a result of our study, non-survivors spent significant more hours in the higher lactate intervals (all intervals >5.0–20.1 mmol/L). Therewith, an overtime elevated serum lactate levels representing an impaired lactate clearance, after initiation of ECLS therapy, was strongly associated with poor outcome during the ICU stay. Previous studies confirmed these findings. An impairment of the lactate clearance significantly correlated with 30-day mortality [26, 32]. But it remains unclear, how an effective lactate clearance looks like under the condition of ECLS therapy and critical ill patients. Due to the small size in this study, the role of liver failure and sepsis, two entities that also contribute to reduced lactate clearance, are not respected closer. Likewise, standardized definitions about lactate clearance should be defined and used in future.

### Limitations

The retrospective nature of this analysis limits the quality of our data. Further, the small sample size from one institution restricts our conclusions. We only collected patients with RCS after cardiac surgery and eCPR due to complicated transcatheter valve implantation/PCI or pulmonary embolism. The small group of patients is very heterogeneous and it is really difficult to understand how much of a role difference in baseline comorbidities, surgical procedure, etc. had in determining mortality. It is conceivable that due to the small group size, the non-significance loses its strength. Likewise, the complex pathology of critical ill patients (a.e. liver failure and sepsis) and additive organ replacement therapies was not respected in detail. Further, the role of the optimal vasopressor regime could not be addressed. There, our findings are consistent with those of recently published studies: the use of epinephrine during VA-ECLS is associated with significant higher mortality [34]. Likewise, the significant greater transfusion burden under VA-ECLS and higher mortality observed in our study was reported for this cohort, recently [35]. Additionally, the unknown role of lactate concentration during critical limb ischemia and resulting lower extremity amputation during VA-ECLS needs to be addressed. However, initial results do not show any significant differences with regard to this parameter [36]. In order to identify ischemia more accurately in the future, a Near-Infrared Spectroscopy (NIRS) Oximetry measurement should be carried out on the affected extremity in addition to the Doppler examination.

## Conclusions

ECLS support is an option for life-threatening conditions as RCS. Based on our results, pre ECLS lactate levels did not differ between survivors and non-survivors. After ECLS initiation, the peak lactate level was a good marker for mortality. Further, elevated lactate levels over time were associated with poor outcome. These findings could be a base to reevaluate the role of lactate and lactate clearance in the first days of ECLS therapy in greater sample sizes in future. With greater sample sizes, the influence of the different causes of the RCS could be better analyzed and interpreted. Then, an adjusted analysis should be performed and include peak lactate as an independent variable along with other pertinent confounders. Additionally, the different timing of VA-ECLS implantation needs to be addressed. Nevertheless, our study supports that hyperlactatemia is a time sensitive emergency under VA-ECLS due to RCS after cardiac surgery, eCPR or complicated transcatheter valve implantation and reversible causes should be cleared as soon as possible to improve patient’s outcome.

## Supporting information

S1 Data (XLSX)

## References

[1] Attanà P, Lazzeri C, Picariello C, Dini CS, Gensini GF, Valente S. Lactate and lactate clearance in acute cardiac care patients. Eur Heart J Acute Cardiovasc Care. 2012 Jun;1(2):115–21. doi: 10.1177/2048872612451168 24062898 PMC3760525

[2] Haas SA, Lange T, Saugel B, Petzoldt M, Fuhrmann V, Metschke M, et al. Severe hyperlactatemia, lactate clearance and mortality in unselected critically ill patients. Intensive Care Med. 2016 Feb;42(2):202–10. doi: 10.1007/s00134-015-4127-0 26556617

[3] Gharipour A, Razavi R, Gharipour M, Modarres R, Nezafati P, Mirkheshti N. The incidence and outcome of severe hyperlactatemia in critically ill patients. Intern Emerg Med. 2021 Jan;16(1):115–23. doi: 10.1007/s11739-020-02337-9 32415561

[4] Minton J, Sidebotham DA. Hyperlactatemia and Cardiac Surgery. J Extra Corpor Technol. 2017;49(1):7–15. 28298660 PMC5347225

[5] Patel B, Diaz-Gomez JL, Ghanta RK, Bracey AW, Chatterjee S. Management of Extracorporeal Membrane Oxygenation for Postcardiotomy Cardiogenic Shock. Anesthesiology. 2021 Sep 1;135(3):497–507. doi: 10.1097/ALN.0000000000003876 34259811

[6] Boeken U, Ensminger S, Assmann A, Schmid C, Werdan K, Michels G, et al. [Use of extracorporeal circulation (ECLS/ECMO) for cardiac and circulatory failure : Short version of the S3 guideline]. Med Klin Intensivmed Notfmed. 2021 Nov;116(8):678–86. 34665281 10.1007/s00063-021-00868-3

[7] Chakaramakkil MJ, Sivathasan C. ECMO and Short-term Support for Cardiogenic Shock in Heart Failure. Curr Cardiol Rep. 2018;20(10):87. doi: 10.1007/s11886-018-1041-4 30116917

[8] Karagiannidis C, Brodie D, Strassmann S, Stoelben E, Philipp A, Bein T, et al. Extracorporeal membrane oxygenation: evolving epidemiology and mortality. Intensive Care Med. 2016 May;42(5):889–96. doi: 10.1007/s00134-016-4273-z 26942446

[9] Alibrahim OS, Heard CMB. Extracorporeal Life Support: Four Decades and Counting. Curr Anesthesiol Rep. 2017;7(2):168–82. doi: 10.1007/s40140-017-0210-0 32288652 PMC7102020

[10] Lunt MJ. Review of duplex and colour Doppler imaging of lower-limb arteries and veins. J Tissue Viability [Internet]. 1999 [cited 2022 Nov 1];9(2):45–55. doi: 10.1016/s0965-206x(99)80013-8 10480971

[11] Vucaj-Cirilović V, Nikolić O, Petrović K, Govorcin M, Hadnadev D, Stojanović S. [Basic characteristics of duplex sonography in the assessment of lower limb arterial circulation]. Med Pregl [Internet]. 2006 [cited 2022 Nov 1];59(5–6):287–90. doi: 10.2298/mpns0606287v 17039916

[12] Lee JH, Hwang SY, Kim HR, Kim YW, Kang MJ, Cho KW, et al. Effectiveness of the sequential organ failure assessment, acute physiology and chronic health evaluation II, and simplified acute physiology score II prognostic scoring systems in paraquat-poisoned patients in the intensive care unit. Hum Exp Toxicol. 2017 May 1;36(5):431–7. doi: 10.1177/0960327116657602 27387349

[13] Laupland KB, Zygun DA, Doig CJ, Bagshaw SM, Svenson LW, Fick GH. One-year mortality of bloodstream infection-associated sepsis and septic shock among patients presenting to a regional critical care system. Intensive Care Med. 2005 Feb;31(2):213–9. doi: 10.1007/s00134-004-2544-6 15666140

[14] Lee HS, Kim HS, Lee SH, Lee SA, Hwang JJ, Park JB, et al. Clinical implications of the initial SAPS II in veno-arterial extracorporeal oxygenation. J Thorac Dis. 2019 Jan;11(1):68–83. doi: 10.21037/jtd.2018.12.20 30863575 PMC6384379

[15] Shah AH, Puri R, Kalra A. Management of cardiogenic shock complicating acute myocardial infarction: A review. Clin Cardiol. 2019 Apr 1;42(4):484. doi: 10.1002/clc.23168 30815887 PMC6712338

[16] Bakker J, Postelnicu R, Mukherjee V. Lactate: Where Are We Now? Crit Care Clin. 2020 Jan 1;36(1):115–24. doi: 10.1016/j.ccc.2019.08.009 31733674

[17] Schorr C a, Dellinger RP. The Surviving Sepsis Campaign: past, present and future. Trends Mol Med. 2014 Apr;20(4):192–4. doi: 10.1016/j.molmed.2014.02.001 24698888

[18] Jansen TC, Van Bommel J, Schoonderbeek FJ, Sleeswijk Visser SJ, Van Der Klooster JM, Lima AP, et al. Early lactate-guided therapy in intensive care unit patients: a multicenter, open-label, randomized controlled trial. Am J Respir Crit Care Med. 2010 Sep 15;182(6):752–61. doi: 10.1164/rccm.200912-1918OC 20463176

[19] Li J, Long C, Lou S, Hei F, Yu K, Wang S, et al. Venoarterial extracorporeal membrane oxygenation in adult patients: predictors of mortality. Perfusion. 2009 Jul;24(4):225–30. doi: 10.1177/0267659109348725 19808747

[20] Chen JS, Ko WJ, Yu HY, Lai LP, Huang SC, Chi NH, et al. Analysis of the outcome for patients experiencing myocardial infarction and cardiopulmonary resuscitation refractory to conventional therapies necessitating extracorporeal life support rescue. Crit Care Med. 2006;34(4):950–7. doi: 10.1097/01.CCM.0000206103.35460.1F 16484889

[21] Mikkelsen ME, Miltiades AN, Gaieski DF, Goyal M, Fuchs BD, Shah CV., et al. Serum lactate is associated with mortality in severe sepsis independent of organ failure and shock. Crit Care Med. 2009;37(5):1670–7. doi: 10.1097/CCM.0b013e31819fcf68 19325467

[22] Jouffroy R, Saade A, Philippe P, Carli P, Vivien B. Prognostic Value of Blood Lactate and Lactate Clearance in Refractory Cardiac Arrest Treated by Extracorporeal Life Support. Turk J Anaesthesiol Reanim. 2019 Feb 1;47(1):48. doi: 10.5152/TJAR.2018.96992 31276111 PMC6598657

[23] Vincent JL, e Silva AQ, Couto L, Taccone FS. The value of blood lactate kinetics in critically ill patients: a systematic review. Crit Care. 2016;20(1). doi: 10.1186/s13054-016-1403-5 27520452 PMC4983759

[24] Komeyama S, Takagi K, Tsuboi H, Tsuboi S, Morita Y, Yoshida R, et al. The Early Initiation of Extracorporeal Life Support May Improve the Neurological Outcome in Adults with Cardiac Arrest due to Cardiac Events. Intern Med. 2019;58(10):1391–7. doi: 10.2169/internalmedicine.0864-18 30713299 PMC6548935

[25] Khorsandi M, Dougherty S, Bouamra O, Pai V, Curry P, Tsui S, et al. Extra-corporeal membrane oxygenation for refractory cardiogenic shock after adult cardiac surgery: a systematic review and meta-analysis. J Cardiothorac Surg. 2017 Jul 17;12(1). doi: 10.1186/s13019-017-0618-0 28716039 PMC5512816

[26] Mungan I, Kazancl D, Bektaş Ş, Ademoglu D, Turan S. Does lactate clearance prognosticates outcomes in ECMO therapy: a retrospective observational study. BMC Anesthesiol. 2018 Oct 24;18(1). doi: 10.1186/s12871-018-0618-1 30355289 PMC6201528

[27] Lee SG, Song J, Park DW, Moon S, Cho HJ, Kim JY, et al. Prognostic value of lactate levels and lactate clearance in sepsis and septic shock with initial hyperlactatemia: A retrospective cohort study according to the Sepsis-3 definitions. Medicine. 2021 Feb 19;100(7):e24835. doi: 10.1097/MD.0000000000024835 33607851 PMC7899836

[28] Filho RR, Rocha LL, Corrêa TD, Souza Pessoa CM, Colombo G, Cesar Assuncao MS. Blood Lactate Levels Cutoff and Mortality Prediction in Sepsis-Time for a Reappraisal? A Retrospective Cohort Study. Shock. 2016 Oct 1;46(5):480–5. doi: 10.1097/SHK.0000000000000667 27380535 PMC5058781

[29] Levraut J, Ichai C, Petit I, Ciebiera JP, Perus O, Grimaud D. Low exogenous lactate clearance as an early predictor of mortality in normolactatemic critically ill septic patients. Crit Care Med. 2003 Mar 1;31(3):705–10. doi: 10.1097/01.CCM.0000045561.85810.45 12626973

[30] Hamiko M, Slottosch I, Scherner M, Gestrich C, Wahlers T, Putensen C, et al. Timely extracorporeal membrane oxygenation assist reduces mortality after bypass surgery in patients with acute myocardial infarction. J Card Surg. 2019 Nov 1;34(11):1243–55. doi: 10.1111/jocs.14258 31523850

[31] Hernandez G, Bellomo R, Bakker J. The ten pitfalls of lactate clearance in sepsis. Intensive Care Med. 2019 Jan 1;45(1):82. doi: 10.1007/s00134-018-5213-x 29754310 PMC6334727

[32] Slottosch I, Liakopoulos O, Kuhn E, Scherner M, Deppe AC, Sabashnikov A, et al. Lactate and lactate clearance as valuable tool to evaluate ECMO therapy in cardiogenic shock. J Crit Care. 2017 Dec 1;42:35–41. doi: 10.1016/j.jcrc.2017.06.022 28672145

[33] Chen H, Gong SR, Yu RG. Association between normalized lactate load and mortality in patients with septic shock: an analysis of the MIMIC-III database. BMC Anesthesiol. 2021 Dec 1;21(1). doi: 10.1186/s12871-021-01239-3 33435876 PMC7802303

[34] Massart N, Mansour A, Ross JT, Ecoffey C, Aninat C, Verhoye JP, et al. Epinephrine administration in venoarterial extracorporeal membrane oxygenation patients is associated with mortality: a retrospective cohort study. ESC Heart Fail. 2021 Aug 1;8(4):2899. doi: 10.1002/ehf2.13370 33963814 PMC8318444

[35] Guimbretière G, Anselmi A, Roisne A, Lelong B, Corbineau H, Langanay T, et al. Prognostic impact of blood product transfusion in VA and VV ECMO. Perfusion. 2019 Apr 1;34(3):246–53. doi: 10.1177/0267659118814690 30444173

[36] Hu S, Lu A, Pan C, Zhang B, ling Wa Y, Qu W, et al. Limb Ischemia Complications of Veno-Arterial Extracorporeal Membrane Oxygenation. Front Med (Lausanne) [Internet]. 2022 Jul 15;9:938634. 35911410 10.3389/fmed.2022.938634 PMC9334727

